# The Role of the Thalamus in Declarative and Procedural Linguistic Memory Processes

**DOI:** 10.3389/fpsyg.2021.682199

**Published:** 2021-09-23

**Authors:** Bruce Crosson

**Affiliations:** ^1^ Center for Visual and Neurocognitive Rehabilitation, Atlanta VA Medical Center, Decatur, GA, United States; ^2^ Department of Neurology, Emory University, Atlanta, GA, United States; ^3^ Department of Psychology, Georgia State University, Atlanta, GA, United States

**Keywords:** thalamus, procedural memory, declarative memory, language, word finding, syntax, grammar, thalamic connectivity

## Abstract

Typically, thalamic aphasias appear to be primarily lexical-semantic disorders representing difficulty using stored declarative memories for semantic information to access lexical word forms. Yet, there also is reason to believe that the thalamus might play a role in linguistic procedural memory. For more than two decades, we have known that basal ganglia dysfunction is associated with difficulties in procedural learning, and specific thalamic nuclei are the final waypoint back to the cortex in cortico-basal ganglia-cortical loops. Recent analyses of the role of the thalamus in lexical-semantic processes and of the role of the basal ganglia in linguistic processes suggest that thalamic participation is not simply a matter of declarative vs. procedural memory, but a matter of how the thalamus participates in lexical-semantic processes and in linguistic procedural memory, as well as the interaction of these processes. One role for the thalamus in accessing lexical forms for semantic concepts relates to the stabilization of a very complex semantic-lexical interface with thousands of representations on both sides of the interface. Further, the possibility is discussed that the thalamus, through its participation in basal ganglia loops, participates in two linguistic procedural memory processes: syntactic/grammatical procedures and procedures for finding words to represent semantic concepts, with the latter interacting intricately with declarative memories. These concepts are discussed in detail along with complexities that can be addressed by future research.

## Introduction and Foundational Concepts

The thalamus is a group of nuclei deep within each cerebral hemisphere, abutting the third ventricle at its medial aspect. It once was thought to be a set of nuclei relaying information between the periphery and the cerebral cortex (Sherman and Guillery, 2006). Based on this viewpoint, the fact that ischemic lesions in the polar artery territory and small hemorrhages in the pulvinar of the left thalamus consistently cause aphasia (Crosson, 1992, 2013) was a conundrum when three-dimensional structural brain imaging first made *in vivo* observation of this phenomenon possible late in the last century. The fact that most such lesions were completely (e.g., Crosson, 1984; Nadeau and Crosson, 1997) or nearly completely (e.g., Crosson et al., 1997) confined to the thalamus of the language dominant (left) hemisphere indicated that the thalamus did more than just relay information or commands between the thalamus and the periphery: i.e., it played some central role in language.

As cases of thalamic aphasia accumulated, a definition of thalamic aphasia began to emerge during the latter portion of the twentieth century. In his review of the literature, Crosson (1984) described four cardinal symptoms of thalamic aphasia: (1) Frequent paraphasias (word substitutions) in spoken language and naming, most frequently semantically related to the target word, (2) jargon in narrative discourse, (3) comprehension less impaired than spoken output, and (4) minimally impaired or unimpaired repetition. Although Cambier et al. (1982) had included these characteristics in their definition of thalamic aphasia, they included other symptoms, such as reduced vocal volume, that could not be confirmed for many cases. Aphasias resulting from hemorrhagic lesion of the dominant thalamus generally conform well to Crosson’s (1984) definition of thalamic aphasia, with the exception that milder cases may not demonstrate jargon. It should be noted that the hemorrhagic cases tended to involve the posterior thalamus, including the pulvinar. Cases of aphasia resulting from dominant thalamic infarction, mostly anterior to the pulvinar, show more variability in symptoms with only about half conforming to the symptom pattern described above. In particular, only about one third of the dominant polar artery infarcts conformed to the constellation of symptoms commonly shown with hemorrhagic lesions (Crosson, 1992).

The variability in symptoms in cases of aphasia with dominant thalamic infarction raises an important question: Is it possible that different thalamic nuclei and tracts might play varying roles in language? Such a conclusion would be consistent with the fact that different thalamic nuclei have unique relationships with different cortical regions (Parent, 1996; Jones, 2007; Waxman, 2017). Further, systematic work from Murray Sherman’s laboratory in the last two decades has convincingly indicated that the higher order thalamic relays are capable of transferring information from one cortical region to another (see Sherman and Guillery, 2006; Usrey and Sherman, 2019 for reviews), and detailed models of how basal ganglia loops support motor functions (e.g., Nambu et al., 2000; Nambu, 2003) were applied explain how lesion and functional imaging studies indicate some role for the basal ganglia in language (e.g., Crosson et al., 2007), with thalamic nuclear regions acting as the gateway from the basal ganglia to the cortex. The former data on thalamic relays has recently been applied to explain lexical-semantic functions in language production (Crosson, 2021) that compose a linguistic branch of declarative memory. The latter conceptualization of basal ganglia functions in language has been followed by recent exciting analysis of basal ganglia functions in language (Copland et al., 2021; Nadeau, 2021) that have implications for linguistic procedural memory functions.

The purpose of the current article is to address the following question: Can the declarative vs. procedural memory distinction explain some of the variability seen in thalamic aphasias, particularly evident in thalamic infarctions? In part, this question is motivated by anatomical considerations. The basal ganglia have long been known to be involved some aspects of procedural memory (e.g., Heindel et al., 1988, 1989). The posterior portion of the polar artery territory subsumes the anterior portion of the ventral lateral nucleus (VLa) which receives projections from the globus pallidus, the output nucleus for the basal ganglia (Jones, 2007), and lesion of the dominant polar artery territory is invariably accompanied by aphasia (Crosson, 1992). The dominant pulvinar, where hemorrhage or infarction also causes aphasia is not connected to the basal ganglia, but it is prolifically connected to perisylvian language regions (Crosson, 1992; Jones, 2007).

The author will explore the question just raised by addressing the following issues. First, two foundational issues will be covered: The first is to review the declarative vs. procedural memory distinction within the context of different forms of memory, and the second is the relevance of thalamic connectivity to the declarative vs. procedural memory distinction. With this preparation, we will then be able to explore how the thalamus supports declarative memory functions in language. This discussion will be followed by consideration of possible roles the dominant thalamus (and basal ganglia) might play in language and whether these roles might be a form of procedural memory. Literature on thalamic aphasia, functional imaging of the thalamus during language tasks, and linguistic effects of thalamic stimulation are reviewed in the sections on linguistic declarative and procedural memory.

### Declarative, Nondeclarative, and Procedural Memory

In his 1949 book *The Concept of Mind*, the philosopher Gilbert Ryle described two forms of memory: “knowing that” (declarative memory) and “knowing how” (procedural memory; Ryle, 1949). As this distinction made its way into the psychological and neuroscience literatures, knowledge about the world (semantic memory) and memory for events (episodic memory) were lumped under declarative memory, while skills, priming, and classical conditioning were often clustered under procedural memory (Squire, 1987). However, the field gradually gravitated towards the position that procedural memory involved learning and performance of habits and skills and that priming and classical conditioning did not belong under the heading of procedural memory. Rather, all these latter forms of memory were subsumed under the broad classification of nondeclarative memory (e.g., Squire and Dede, 2015).

From an anatomic standpoint, medial temporal structures (entorhinal cortex, hippocampus) and diencephalon (midline and dorsal medial thalamus, mammillary bodies) are involved in instantiation of declarative memories. The role of the basal ganglia (striatum, globus pallidus) in instantiation of procedural memories has been known for some time based on studies of the basal ganglia disorders, such as Huntington’s disease (Martone et al., 1984; Heindel et al., 1989). Parkinson’s disease is also a basal ganglia disorder, but the findings are both more plentiful and more complicated. Parkinsonian patients frequently show both procedural and declarative memory deficits (Allain et al., 1995; Thomas et al., 1996), though such findings may be task dependent (Harrington et al., 1990). It is worth noting that Parkinsonian patients with declarative memory deficits have reduced thickness in the CA1 stratum pyramidale subfield of the hippocampus (La et al., 2019), which is consistent with anatomical correlates of declarative memory mentioned above.

The concepts of declarative and procedural memory have been applied to the realm of language. The knowledge of words and their associations with objects, actions, and characteristics form a lexicon, which has been deemed a form of declarative memory. The lexicon also subsumes the meaning of suffixes, prefixes, and idiomatic phrases (Ullman, 2004). The knowledge of how to inflect and order those words into sentences, along with function words such as articles, prepositions, and auxiliary verbs, constitutes grammar (syntax and morphology), which can be considered a type of procedural memory. Like other forms of procedural memory, grammar is learned through repeated experience and usually is invoked automatically (Ullman, 2004).

For the purposes of this article, it is also useful to make a distinction between learning and memory. Learning refers to the process of acquiring new information or new skills. Memory, on the other hand refers, refers to the persistence of the learned information or skill in the brain for utilization at a later time (Squire, 1987). Ullman’s (Ullman et al., 1997; Ullman, 2004) classification of syntax and related grammatical procedures falls under this latter distinction, that of already learned skills. Hence, this article will deal primarily with access or utilization of already acquired linguistic information (e.g., semantic-lexical associations) or already learned linguistic skills (e.g., syntax and morphology). In addition, we will discuss the possibility that word-finding involves the use of both semantic-lexical associations and learned procedures that enhance the accuracy and efficiency of pairing semantic concepts with a word to represent them.

### Thalamic Connectivity in Declarative and Procedural Memory

**Figure 1:**
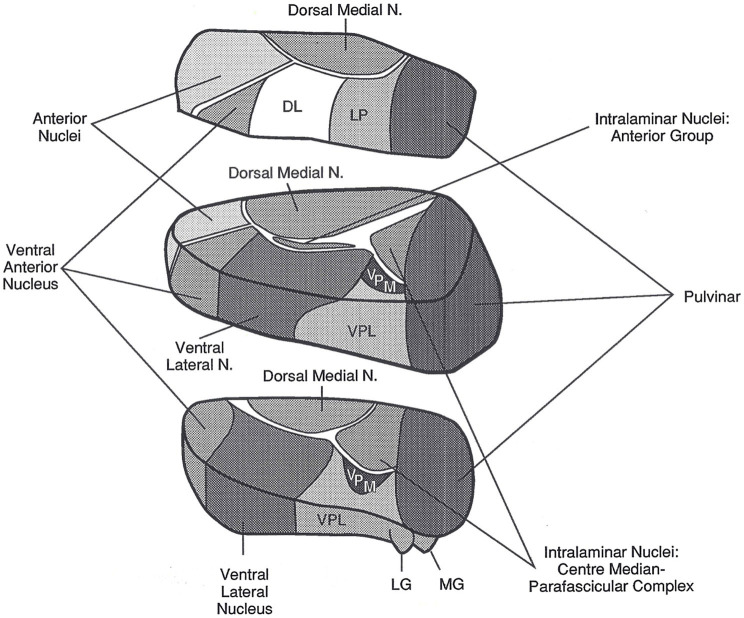
The left thalamus. This superolateral view of the left thalamus in three blocks shows its major nuclei. The current article focuses on the pulvinar, the large nucleus in the posterior most portion of the thalamus, and on the VA/VLa region, consisting of the ventral anterior nucleus (VA) and the anterior portion of the ventral lateral nucleus (VLa). The internal medullary lamina are the “white” bands of fibers separating many of the nuclei. DL, dorsal lateral nucleus; LG, lateral geniculate nucleus; LP, lateral posterior nucleus; MG, medial geniculate nucleus; N, nucleus; VPL, ventral posterior lateral nucleus; VPM, ventral posterior medial nucleus. Reprinted from Crosson (1992). Copyright 1992, with permission from Guilford Press.

Before proceeding with this discussion, it is critical to discuss thalamic connectivity because the arguments for considering the proposition that the thalamus might be involved in both declarative and procedural memory processes for language relies, in part, on anatomical considerations. The thalamus (Figure 1) is a cluster of nuclei in the dorsal diencephalon deep within each cerebral hemisphere. The medial wall of the thalamus lies adjacent to the third ventricle, and in humans, the left and right thalami are often bridged across a portion of the third ventricle by a gray matter mass referred to as the massa intermedia. The thalamus is often referred to in the neuroimaging literature as if it is a single, unitary structure with a simple monolithic contribution to behavior and cognition. Nothing could be further from the truth. The thalamus is divided into several distinct nuclei (Figure 1), and further into subnuclear areas, and the contributions of each nucleus and its constituent areas to behavior and cognition are closely related to the cortical and subcortical structures to which they are connected (Sherman and Guillery, 2006).

In the left thalamus, infarcts in the polar artery territory and hemorrhages in the posterior thalamus almost always cause aphasia (Crosson, 1992, 2013), indicating these thalamic regions are involved in language. For the purpose of identifying language functions to which the thalamus might contribute, it is ideal that lesions be limited to the thalamus. Though this is not always the case, it happens frequently enough to clearly implicate the thalamus in language functions (Crosson, 1984, 1992). The polar artery infarcts usually subsume the ventral anterior nucleus (VA), but also may extend into an adjacent thalamic white matter band known as the internal medullary lamina as well as into the adjacent anterior portion of the VLa (e.g., see Nadeau and Crosson, 1997). The globus pallidus is the output nucleus of the basal ganglia and projects fibers into this region of the thalamus. There has been some disagreement whether the termination of these pallidal projections is on neurons belonging to VA or VLa, though more recent literature seems to be gravitating toward the position that they belong to VLa (Jones, 2007). Nonetheless, since the language literature often assumes the target is VA, and to avoid confusion, the current article will refer to the area subsumed by polar artery lesions as VA/VLa.

Posterior hemorrhagic lesions in the dominant (left) thalamus that cause aphasia usually involve the pulvinar (e.g., Crosson et al., 1986, 1997; Crosson, 1992). The human pulvinar occupies a proportionately larger part of the thalamus than in other primates (Chalfin et al., 2007). Hence, it is fitting that it be involved in language, since complex language is the feature that most highly distinguishes humans from other primates. While it may be difficult to localize the part of the pulvinar involved in language from the hemorrhagic lesions that cause aphasia, interruption of naming by intra-operative electrical stimulation of the pulvinar has implicated the anterior superior lateral pulvinar (Ojemann, 1977). Left pulvinar stimulation affects naming with a greater frequency than left VLa stimulation (Crosson, 1984). It is worth noting that lesions in the dominant paramedian artery territory occasionally, though far from always, cause aphasia (e.g., Crosson, 1992; Nadeau and Crosson, 1997). These lesions are centered in the dorsal medial nucleus, but also can involve the adjacent internal medullary lamina.

**Figure 2:**
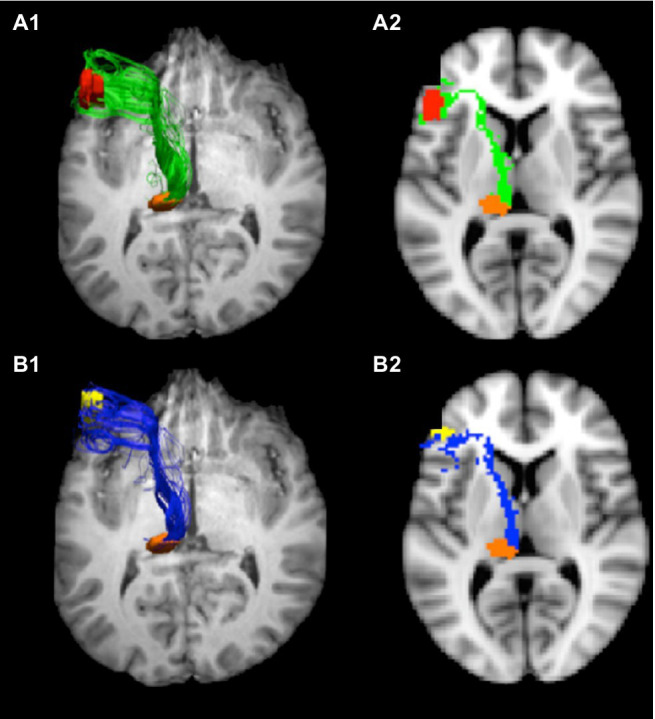
Tracts from Broca’s area to pulvinar. Connections from Broca’s area enter the internal medullary lamina between the anterior and ventral anterior nuclei, where some fibers enter the ventral anterior nucleus and others course *via* the internal medullary lamina to the pulvinar. These images show the tracts from pars opercularis **(A1,A2)** and pars triangularis **(B1,B2)** to the pulvinar. The images on the left **(A1,B1)** represent 3-D views of the tracts overlaid on an axial image ventral to the tracts, and the images on the right are 2-D rendering of the tracts through a single slice (*Z*=38). Adapted from Bohsali et al. (2015). Copyright 2015 with permission from Elsevier.

Nuclei involved in thalamic aphasia are connected to perisylvian language cortices in the language dominant hemisphere. Bohsali (formerly Ford) and her colleagues have traced fibers from Broca’s area (left pars triangularis and pars opercularis) to the thalamus and putamen using mixture of Wisharts probability distributions and probabilistic tractography of diffusion-weighted magnetic resonance images (MRI; Ford et al., 2013; Bohsali et al., 2015). The fiber bundles headed for the thalamus (Figure 2) course medially, forming a vertical sheet, as they emerge from pars triangularis and pars opercularis. As they approach the anterior forceps of the corpus callosum, fibers turn posteriorly and enter the anterior limb of the internal capsule between the caudate head and putamen. After reaching the genu of the internal capsule, the sheet of fibers enters the thalamus at the internal medullary lamina, between the anterior nucleus and the ventral anterior nucleus. Some of these fibers terminate in the ventral anterior nucleus while others travel posteriorly *via* the internal medullary to terminate in the pulvinar (Bohsali et al., 2015). The verticality of this sheet of fibers conforms to the orientation of the internal medullary lamina within the thalamus. As noted above, dominant polar artery infarcts affecting VA/VLa and dominant posterior thalamic hemorrhages including parts of the pulvinar are commonly implicated in thalamic aphasias; thus, it has been suggested that both locations may be involved in language functions (Crosson, 1992, 2013).

The fibers entering the putamen do so at its anterior aspect (Ford et al., 2013). However, recent work by Roberts (2017) suggests that some fibers from pars triangularis and pars opercularis course superiorly over the putamen entering it even at its posterior aspect. These connections, of course, indicate that these components of Broca’s area are likely part of cortico-striato-pallido-thalamo-cortical loops back to Broca’s area. In a functional imaging study, Crosson et al. (2003) also implicated a different basal ganglia loop in word generation. This loop involved cortex straddling pre-supplementary motor area (pre-SMA) and Brodmann’s area 32, the dorsal caudate nucleus, and the ventral anterior nucleus of the thalamus.

To summarize, two areas of the dominant thalamus that are strongly associated with thalamic aphasia have been shown to be connected to Broca’s area. These are VA/VLa and the pulvinar. The posterior portion of VA/VLa receives input from the globus pallidus, and the neurons receiving this input may belong more to the VLa than the VA (Jones, 2007). The region of VA/VLa appears to be involved in a basal ganglia loop involving cortex straddling pre-SMA and Brodmann’s area 32 as well as the dorsal caudate nucleus (Crosson et al., 2003). Since the putamen receives input from Broca’s area (Ford et al., 2013; Roberts, 2017), it is likely that VA/VLa is also involved in a basal ganglia loop with Broca’s area. The pulvinar, on the other hand, is not connected with the basal ganglia. Like other frontal areas (Goldman-Rakic and Porrino, 1985), Broca’s area projects to the pulvinar (Bohsali et al., 2015). This medial division of this nucleus also receives rich input from multiple areas of the temporal and posterior parietal lobes (Jones, 2007), which have rich language functions in humans. In the remaining sections of this paper, we will make a case that the pulvinar is involved in lexical-semantic functions which are declarative in nature and explore the possibility that the VA/VLa is involved in some procedural memory functions for language.

## Declarative Lexical-Semantic Functions and the Thalamus

As previously noted, Crosson (1984) reviewed the literature on thalamic aphasia and found 4 cardinal symptoms: (1) word substitutions in spoken language, primarily semantically in nature, (2) jargon in narrative discourse, consisting of words from the patient’s native language, (3) comprehension less impaired than spoken output, and (4) minimally impaired repetition. Aphasias resulting from hemorrhagic lesion of the posterior dominant thalamus conform well to these criteria. It should be noted that the hemorrhagic cases usually involved the posterior thalamus, including the pulvinar. Cases of aphasia resulting from dominant thalamic infarction show more variability in symptoms, suggesting that subtle differences in location within the thalamus could contribute to variability of the symptom pattern for cases of thalamic infarction. For example, the antero-medial edge of polar artery lesions may impinge on fibers of the anterior portion of the internal medullary lamina between VA and the anterior nuclear group, which connect Broca’s area and the pulvinar, disrupting communication between these two regions. On the other hand, the posterior edge of polar artery lesions may impinge on VLa, which receives input from the basal ganglia *via* the globus pallidus. Hence, variability in the size and location of polar artery lesions affects whether they interrupt communication between language areas and the pulvinar and/or communication between the basal ganglia and VA/VLa. Should these two patterns of thalamic connectivity serve different functions, then variability in the interruption of these connectivities could explain the variability in language symptoms after dominant polar artery infarction. Such observations about connectivities of the thalamus and cognitive symptoms are not new; in particular, von Cramon et al. (1985) made similar observations regarding the necessity of infarcts interrupting the mammillothalamic tract to cause profound memory impairment.

In any event, the most common symptom of thalamic aphasia is word-finding difficulty with semantic paraphasias, deteriorating to jargon in more severe cases. Adherence of posterior dominant thalamic hemorrhages to the criteria for thalamic aphasia (Crosson, 1984) indicates that this symptom pattern occurs with involvement of the pulvinar. Since the ability to match a lexical item (word) to a concept is declaring that the lexical form represents the concept, the act of word finding can be considered a form of declarative memory, though we will later entertain the notion that word finding also involves a procedure for pairing words and concepts.

All this being said, however, it would be inherently unsatisfying to state that the dominant pulvinar is playing a role in this form of declarative memory without digging more deeply. The important question is why patients with thalamic aphasia are unable to pair a correct word with a concept when they are speaking. Has the semantic concept somehow become fragmented? Does the lexical form no longer exist or has it become weakened? Or, is there a problem accessing intact words given an intact semantic concept? To the knowledge of this author, the study by Raymer et al. (1997) still provides the most complete answer to these questions. These authors assessed two patients with thalamic aphasia (a 45-year-old female and a 59-year-old male) 5 and 4months post-onset, respectively. The patients named 120 objects using three subtests on the Florida Semantics Battery: (1) oral picture naming, (2) written picture naming, and (3) naming to auditory definition. The use of this three-subtest combination was designed to ascertain whether naming difficulties can be attributed to modality of input (visual picture vs. auditory definition) or mode of output (written word vs. spoken word). Since the patients showed difficulty on all three naming subtests relative to age, gender, and education-matched controls, their naming deficits were deemed to be independent of modality of input or mode of expression. Both patients had greater difficulty with low frequency than medium or high frequency words. Flawless oral reading and nearly flawless writing to dictation of the 120 words indicated that the spoken and written word forms were available to the patients. Unimpaired matching of auditory and written words to pictures indicated that the relationship between words and visual concepts was understood. The authors concluded that patients’ deficits involved a defect in accessing word forms from semantic concepts, to which we refer as a semantic-lexical deficit.

Of interest is that the deficits in these patients were so similar while the location of the lesions within the thalamus was quite different. These lesions were mapped onto atlas templates by Nadeau and Crosson (1997). The woman had a left polar artery infarct centered in the VA but also affecting the VLa and the adjacent section of the internal medullary lamina. The gentleman had a left paramedian artery infarction affecting the dorsal medial nucleus, the centromedian nucleus, the parts of the VLa, and the internal medullary lamina. The degree of lesion overlap in thalamic nuclei between the subjects was minimal, but the lesions did appear to involve fibers connecting anterior language areas with the pulvinar. Hence, it is not surprising that their typical thalamic aphasias were quite similar to aphasias resulting from lesions in the posterior thalamus, including the pulvinar (e.g., Crosson et al., 1986). From this perspective, it is of interest to note that lesion of the internal medullary lamina in Raymer and colleagues’ patients would have interrupted fiber the pathway between Broca’s area and the left pulvinar as mapped by Bohsali et al. (2015), albeit at slightly different locations in its anterior–posterior trajectory. In other words, an anatomic commonality between the polar and paramedian artery lesions of Raymer and colleagues’ cases and more posterior lesions causing thalamic aphasia is interruption of the information transmission pathway between Broca’s area and the pulvinar. As applied to the current conundrum, adherence to the symptom cluster of thalamic aphasia as defined by Crosson (1984) of lesions with different thalamic locations may be due to the fact that lesions at these different locations interrupt information transfer between Broca’s area and the pulvinar.

This observation of a common anatomic substrate for polar artery, paramedian artery, and posterior thalamic lesions is important, but it does not reveal what function this substrate performs. In 2013, Crosson noted cortico-thalamo-cortical circuits as one among four thalamic mechanisms potentially affecting language, specifically word retrieval. He cited evidence from Sherman and colleagues (e.g., Sherman and Guillery, 2006; Theyel et al., 2010; Usrey and Sherman, 2019) noting that these cortico-thalamo-cortical circuits pass information from one cortical area to another. The conundrum that Crosson raised is why cortico-thalamo-cortical circuitry was necessary when direct cortico-cortical connections existed and could pass the information on more efficiently (i.e., with a single intervening synaptic interface as opposed to two). In a more recent article Crosson et al., 2021, he used information from Kawaguchi’s work (Morishima et al., 2011; Kawaguchi, 2017) about corticothalamic neurons and insights from parallel distributed processing (PDP) models (Rummelhart et al., 1986; Nadeau, 2021) to suggest the role cortico-thalamo-cortical circuitry plays in word finding.

The complexity of converting semantic information into a lexical item for expression of the semantic concept should not be under-estimated. One study of English-speaking college students suggested that the average number of words known was around 17,000 (D’Anna et al., 1991). The number of semantic concepts (objects, actions, attributes) known must be in the thousands as well. Searching for a single correct word for a semantic concept by trying to find one word amongst 17,000 lexical competitors would be inefficient and time consuming without a learned procedure to impose efficiency upon the process. Essentially, PDP models suggest that word finding is an iterative process that zeroes in on the best word choice, rather than a simple linear progression accomplished in one pass (Rummelhart et al., 1986; Nadeau, 2012). This iterative process takes place by proceeding through successively smaller lexical-semantic neighborhoods until a correct choice can be made from a limited number of competitors. For example, if one is asked to name a picture of a German Shepherd, the first pass of iterative processing might be to narrow down the choices to the lexical-semantic neighborhood of animals (cutting down the 17,000 potential choices considerably) and in the second pass to four-legged mammals, then from four-legged mammals to canines, then from canines to (domestic) dogs, and then finally from dogs to German Shepherd. At each step of the way, the narrowing down of the lexical-semantic choice is compared to the original semantic concept to ensure that the concept is consistent with the lexical-semantic choice. In other words, a continuing mental record of the semantic concept is necessary to undergird the word selection process.

The important insight from Kawaguchi’s (2017) work was that corticocortical neurons change firing patterns rapidly, whereas corticothalamic neurons change firing patterns slowly. Hence, Crosson (2021) reasoned that while corticocortical processes rapidly and iteratively convert a semantic concept to a word choice, cortico-thalamo-cortical circuits maintain a semantic representation that can be compared to choices made at each step of retrieving the best lexical representation to ensure that the lexical decisions are associated with the semantic concept. In one of our picture-naming studies (Wierenga et al., 2008), it took younger participants (20–34years old) an average of 1,400+ ms from first presentation of the picture to speaking the word representing the object in the picture, and it took older participants (68–84years old) an average of 1,600+ ms to do the same. This amount of time would not be necessary for a simple linear transmission of information from one cortical processor to the next, since such transmission can occur on the order of milliseconds. Rather, the amount of time necessary to produce a name for a picture indicates the more complex nature of the underlying neural processes: i.e., the iterative neural transmissions necessary to progress from a semantic concept through successively smaller lexical-semantic neighborhoods and finally to a word choice.

What happens in this model if the cortico-thalamo-cortical circuits are interrupted by lesion? Without the feedforward and feedback processes afforded by maintenance of the semantic concept, the iterative processing is interrupted, and a choice is made from the semantically organized lexical neighborhood without the ability to precisely narrow down the word choice through iterative processing. This analysis explains why thalamic aphasias, especially acutely, make semantically related, but incorrect word choices that can deteriorate into semantic jargon. Indeed, particularly in the first few days post onset, semantic paraphasias in thalamic aphasia may reflect choice from a large lexical-semantic neighborhood at a relatively early stage in narrowing down the choices. For example, in repeating a short story about a ship hitting a mine near Liverpool from the original Wechsler Memory Scale (Wechsler, 1945), Crosson et al.’s (1986) thalamic aphasia case referred to ordnance, a supraordinate category subsuming mine, indicating that the word search was truncated at the supraordinate category before performing a search of it.

A cluster of functional MRI (fMRI) studies also has some bearing on the role of the thalamus in semantic processing. Kraut et al. (2002a,b) presented two features for potential objects, and participants responded when the two features could be combined to make an object and withheld responding when they could not. For example, when given “desert” and “humps,” the participant should activate the concept of camel and respond by pressing a button, but when given “bullets” and “milk” they should withhold responding because these features cannot be combined to make an object. In one study (Kraut et al., 2002b), the features were presented as words, and in the other study (Kraut et al., 2002a), the features were presented as pictures. When the features were presented as words, an area designated as the left “dorsal thalamus” showed increased activity when the features were recognized as forming an object. When features were presented as pictures, both the left and right “dorsal thalamus” showed increased activity. Kraut et al. (2003) used the version of the task where features were presented as words with a different set of subjects and employed refined analytics to make inferences about the function of different areas of activity increase. Specifically, they used the rise time, peak time, and fall time of the hemodynamic responses to infer the role of activated areas on trials where features combined to constitute a real object. They found two areas of activity in the thalamus, pulvinar and dorsal medial nucleus, with different time signatures. An area including the dorsal medial nucleus demonstrated hemodynamic response curves that indicated involvement relatively early in the task, like pre-SMA. This region was thought to be involved in designating or refining search criteria. In other words, the dorsal medial thalamus was thought to be involved in the process of word finding. However, the pulvinar showed the slowest developing hemodynamic response, indicating it was involved in binding the two features into a real object as the concluding step of the search. The important point for current purposes is that the pulvinar was facilitating the maintenance and integration of semantic information.

Although the emphasis for the current discussion is on thalamic contributions to word finding, it is important to emphasize that both cortico-cortical and cortico-thalamo-cortical transmission are intimately intertwined in proceeding from a semantic concept to a lexical representation of that concept in Crosson (2021) model. Hence, interruption of cortico-cortical processing at the semantic-lexical interface could result in similar cognitive manifestations as interruption of cortico-thalamo-cortical processing. In severe cases of cortico-cortical disconnection, however, if the first pass at finding a broad neighborhood of semantically related lexical groups for the desired concept is interrupted, cortico-cortical processing deficits might not produce as many semantically related errors. To the degree that this question of the results of interrupting cortico-cortical processing is dependent upon the lesion literature, it is unfortunately rhetorical. Lesions precise enough only to interrupt cortico-cortical transmission at a specific interface without damaging the cortical mechanisms would seldom, if ever, occur naturally. In other words, investigators could not expect to see the kind of lesions necessary to intricately probe interruption of cortico-cortical processing. While it is theoretically possible to use functional imaging to parse contributions of cortico-cortical from cortico-thalamo-cortical processing, the classical double dissociation necessary to isolate these two forms of processing may be nearly possible to achieve. Specifically, while one might, with a masterful cognitive design, be able eliminate the need for cortico-thalamo-cortical processing, it is difficult to imagine eliminating cortico-cortical processing from language paradigms to help determine its contribution.

In summary, the act of pairing a word with a semantic concept can be considered to rely on linguistic declarative memory since one is declaring that the word represents the concept. The fact that posterior thalamic lesions (hemorrhages) conform well to the syndrome of thalamic aphasia, including its semantic-lexical manifestations, indicates a role for the pulvinar in semantic-lexical processing. Crosson (2021) proposed that this role was making available a stable semantic representation to guide cortico-cortical processing through the iterative word-finding process that narrows down lexical choices to successively smaller neighborhoods of semantically-related lexical items. While small lacunar lesions of the thalamus should make it possible to test theories about the role of dominant thalamic mechanisms in language in lesion paradigms, the lack of access to lesions discrete enough to unravel the nature of cortico-cortical transmission without damage to cortical mechanisms will be a barrier to describing cognitive differences in interruption of cortico-thalamo-cortical vs. cortico-cortical connections during word finding in thalamic lesion studies. Functional imaging studies will face a similar challenge because of the difficulty of dissociating cortico-cortical processing from language tasks to determine the nature of its contribution.

## Linguistic Procedural Memory and the Thalamus

The question of the role of the thalamus in linguistic procedural memory is not as simple as the case for declarative memory. Nonetheless, our exploration of the proposition that thalamic nuclei might be involved in linguistic procedural memory is motivated by anatomic considerations. As already noted, aphasia is nearly always found in cases of dominant polar artery lesions which affect the VA/VLa region (Crosson, 1992) and this region, especially VLa, is a part of cortico-striato-pallido-thalamo-cortical loops (Alexander et al., 1986; Jones, 2007). Aphasia frequently, though not always, occurs after lesion in the dominant paramedian artery territory (Crosson, 1992), and this artery irrigates the dorsal medial thalamus, which also is incorporated into fronto-striato-pallido-thalamo-frontal loops (Alexander et al., 1986; Jones, 2007). Hence, given the involvement of the basal ganglia in procedural memory (e.g., Heindel et al., 1988, 1989), the proposition that these thalamic nuclei may be involved in linguistic procedural memory is undertaken. Specifically, in this section, we raise two possibilities for involvement of thalamic nuclei in procedural memory. First, given Ullman’s (2004) classification of syntactic and other grammatical processes as a form of procedural memory, we explore the possibility that grammatical processes are involved in thalamic aphasia. Second, we raise the possibility that the procedure of successively narrowing down word choices to smaller semantic neighborhoods may constitute a form early and basic linguistic habit instantiated *via* procedural learning, in which the basal ganglia have a role, especially for low frequency words. This latter concept may be controversial, but it raises the possibility that declarative and procedural memory processes in language may not be as easily disentangled as the dichotomous presentation of the concepts suggests.

### Is the Thalamus Involved in Syntax and Grammar?

As previously noted, grammatical (syntactical and morphological) processes have been considered to fall under the rubric of linguistic procedural memory (Ullman et al., 1997; Ullman, 2004). Though rare, grammatical processes have been studied in thalamic aphasia cases, even though results do not allow for a definitive conclusion about potential involvement in these functions. For example, Raymer et al. (1997) noted that their two thalamic aphasia cases showed minimal to no errors in sentence comprehension or syntax production; in other words, these patients showed no syntax-related deficits. At the time of testing, these patients were 5 and 4months post left thalamic infarction. As noted above, lesions were in the polar and paramedian artery territories.

On the other hand, De Witte et al. (2006) performed a more detailed analysis of grammatical comprehension and production for their patient with a bilateral paramedian thalamic infarction. [Bilateral paramedian infarcts happen when both the left and right paramedian arteries are fed by a single twig emerging from the initial segment of one posterior cerebral artery (Caballero, 2010)]. Similar to other thalamic aphasias, this patient made many semantic errors in spoken language, had some impairment of auditory comprehension (especially at the sentence level), but had intact repetition. The assessment of syntactic competence was performed 4weeks after the stroke, 3 to 4months earlier than the assessments reported by Raymer et al. (1997). In a narrative language sample of 318 words, De Witte et al. (2006) described the marked simplification of syntax (e.g., only one subordinate clause in sample), shorter than normal utterances, few conjugated verbs, and a limited number of lexical verbs. The patient had modest impairment in verb and sentence comprehension, but her ability to judge the grammaticality of sentences appeared to be intact. She had significant difficulty on a sentence construction task during which she was asked to describe 20 pictures in a single sentence. Errors included failure to conjugate verbs (i.e., use of infinitives), omission of function words, stereotypic sentence construction, and semantic errors. She made some errors in action naming, mostly semantic. In general, her performance on sentence anagrams (ordering words to make a sentence describing a picture) was intact, with the exception of wh- interrogatives, where she made a significant number of role exchange and role order errors. Hence, at 1month post onset, De Witte et al. (2006) described one patient who had syntactic deficits, most evident in constructing sentences or in a more difficult sentence anagram task. As is characteristic of paramedian artery lesions (unilateral or bilateral), the lesions were primarily in the dorsal medial nucleus, not VA/VLa. As Nadeau (1988) had previously noted syntactic deficits with frontal lesions, the authors attributed the syntactic deficits in their patient to the close connectivity between the dorsal medial nucleus and the frontal lobes. It is also worth noting that the basal ganglia project to the dorsal medial nucleus *via* the globus pallidus and that the dorsal medial nucleus has intimate relationships with every area of prefrontal cortex (Goldman-Rakic and Porrino, 1985; Crosson, 1992).

Since the dorsal medial nucleus and VA/VLa serve as gateways from basal ganglia loops to the cortex, the literature on the effects of basal ganglia lesion and disease is relevant to our discussion. This large volume of literature was recently covered in a superb and comprehensive review by Copland et al. (2021); hence, we will cover the high points of this review in the following paragraphs and refer the reader to the original review for greater detail. A first point of interest is that these authors performed a meta-analysis of neuroimaging data sets with NeuroQuery (Dockès et al., 2020) to predict anatomic correlates of language and processes possibly related to language. This analysis predicted the involvement of the neostriatum (caudate nucleus and/or putamen) along with the inferior frontal and middle temporal gyri in the following processes: language learning, language selection/retrieval, and sentence/sequencing, with medial frontal activity also predicted for the latter two processes. A search for language conflict predicted involvement of the anterior cingulate area, inferior frontal gyrus, superior and middle temporal gyri, and thalamus. Additional review of the literature indicated involvement of cortico-striatal systems in responding to ambiguity and conflict. With thalamic nuclei acting as the gateway for influence of striatal processes on the cortex, it is curious that the thalamus was only found to be involved in the language conflict and not the other searches which implicated the neostriatal portion of basal ganglia loops. To the degree that the Copland (2021) review depended on functional neuroimaging, the lack of significant thalamic signal change in some studies could be related to a number of problems in imaging deep gray matter structures (e.g., Grossman et al., 2009). One such problem is the fact that signal changes related to cognitive/behavioral tasks for BOLD based fMRI, but also for arterial spin labeling perfusion MRI, can be considerably smaller in the thalamus than the cortex. Signal change can be smaller in the striatum as well, but areas of activity change are larger in the striatum than the thalamus (e.g., Boscolo Galazzo et al., 2014). Since most fMRI analysis procedures impose cluster size limitations, the smaller size of thalamic activation clusters could account for the lack of thalamic activation when striatal activation is present. Another potential reason for a lack of significant thalamic activity changes in the presence of striatal activation is that signals may reach the striatum from the cortex but proceed no further through cortico-striato-pallido-thalamo-cortical loops because the signal is suppressed in the striatum. There is a strong network of collateral inhibition in the striatum that might perform such a function (Groves, 1983).

Considering that syntax and other grammatical operations have been declared a part of procedural memory (Ullman, 2004), it is specifically worth noting Copland et al. (2021) review regarding basal ganglia loops in this area. Particularly important is the idea that the basal ganglia are involved in multiple processes through multiple cortico-striato-pallido-thalamo-cortical loops that impact grammatical/syntactic functions (Ghio et al., 2018). Some of the processes may directly involve grammatical procedures, and hence, reflect procedural memory. Other processes may not be grammatical in nature but nonetheless necessary to support processes that are. Some of the functions mentioned by Copland et al. (2021) that evoke basal ganglia activity and can be considered grammatical in nature are: difficulty with inflection of verbs to convey tense (Ullman et al., 1997; Teichmann et al., 2008, 2015), syntactic processing (Teichmann et al., 2008), and grammatical rule processing (Ullman, 2004; Teichmann et al., 2008). Some functions noted by Copland et al. (2021) that support but are not grammatical processing are: working memory (Kemmerer, 1999; Grossman et al., 2002), attention (Grossman et al., 1992; Lee et al., 2003), sequencing words during syntactic processing (Chan et al., 2013), and synchronizing temporal and sequential aspects of syntax comprehension (resulting from a failure to extract predictable cues; Kotz et al., 2009). For purposes of this discussion, it is sufficient to state that there is general agreement in the literature that the basal ganglia contribute to syntactic and related grammatical processes; however, given the number and nature of processes suggested to account for syntactic/grammatical processing deficits in basal ganglia disorders, it seems safe to conclude that we are still in the process of understanding their nature.

Yet, one proposition about basal ganglia contributions to grammatical processing seems worth further consideration. Based on their data, Giavazzi et al. (2018) suggested that the basal ganglia participate in selecting between grammatical alternatives rather than in grammatical processing *per se*. To expand on this concept, for example, it would be consistent with Nambu’s analysis of motor functions (Nambu et al., 2000; Nambu, 2003) and Crosson’s analysis of word finding (Crosson et al., 2003, 2007) to suggest that the basal ganglia boost the activation of the best syntactic frame for expressing an idea while suppressing less appropriate syntactic frames. This concept will be discussed in greater detail as it applies to word finding in the next section.

In summary, syntactic processing problems were found in one of three cases where such functions were assessed after dominant thalamic infarction (Raymer et al., 1997; De Witte et al., 2006). These findings are difficult to interpret because of the small numbers of dominant thalamic lesion cases in which syntactic processing has been assessed. While cases of language deficit after dominant thalamic lesion are not plentiful, thalamic lesion cases are seen periodically in large stroke centers. Syntactic assessment in enough patients to resolve the issue would be welcome. There is agreement that patients with basal ganglia disease have difficulty with syntactic processes; however, our understanding of the nature of these processes may still be in flux. A supraordinate construct that can be applied to selection of motor behaviors and word finding process, as well as to syntactic/grammatical processing, is that the basal ganglia participate in selection of the best motor behavior, word, or grammatical alternative that meets an intended goal in action or communication, while suppressing potential competitor motor behaviors, words, or grammatical alternatives. It is assumed that such a function would be performed *via* basal ganglia loops which use the thalamus as a gateway to influence cortical processing.

### Is There a Procedural Memory Component to Word Finding?

We now turn to what may be a more controversial question: Is there a procedural memory component to word finding? Intuitively, pairing a word with a concept seems to be declarative in nature since we are in a sense declaring that the word represents the concept we are communicating. Yet, this paper has also proposed a procedure by which we find words to represent a concept by iteratively narrowing down semantic neighborhoods of lexical items in which we are conducting a search until there are only a few words from which we make our selection. In this section, we will consider the possibility that this algorithm represents a form of procedural memory that is acquired early in life, in the same organic way that the foundations of syntax and other grammatical operations are acquired. And, like other forms of procedural memory, such searches for words are applied automatically, without conscious planning. We also will implicate a basal ganglia loop in this process that acts through VA/VLa. In the larger context of declarative vs. procedural memory processes, this discussion is important because it casts procedural and declarative memory processes not as isolated from each other, which is what we try to accomplish in experiments to understand these processes. Rather, this discussion portends the importance of the intimate interaction of procedural and declarative processes in every-day functions, which we also must strive to understand.

Hence, while the current paper described picture naming (i.e., declaring the name of an object or action in a picture) as a form of declarative memory, it also described a process, that is a procedure, for narrowing down the lexical search by probing successively smaller semantically organized lexical neighborhoods. There is some evidence that this process for word-finding might involve the basal ganglia, and by implication the thalamic component of cortico-striato-pallido-thalamo-cortical loops. For example, Copland et al. (2000) studied 14 patients with non-thalamic subcortical lesions primarily affecting the basal ganglia and surrounding white matter of the language dominant hemisphere as well as 14 age-matched neurologically normal controls. While, on the average, these subcortical lesion cases would not be considered to have aphasia and did not differ from controls in naming common objects from the *Western Aphasia Battery* (Kertesz, 1982), they were impaired relative to controls in generating animal names and naming the mostly low-frequency items on the *Boston Naming Test* (Kaplan et al., 1983). The lack of a clinically significant language disorder (aphasia) on the average in this subcortical lesion group suggests that impairment of search procedures during word retrieval (as opposed to lexical or semantic representations *per se*) might account for their word-finding difficulties.

Some evidence from the neuroimaging literature implicates VA/VLa and its role in one basal ganglia loop in word finding. Crosson et al. (2003) had healthy young participants perform four language fluency tasks during fMRI: generation of members for categories with a relatively large number of items, generation of members for categories having a relatively small number of items, generation of words rhyming with a given word, and generating pseudowords given beginning and ending consonant blends. For each word generation task compared to visual fixation, significant activity increases were seen in a basal ganglia loop involving cortex straddling the pre-SMA and the paracingulate gyrus, the dorsal caudate nucleus, and VA/VLa thalamic region. (The pallidal component of this loop could not be visualized, likely due to interference from manganese deposits, which have an affinity for the globus pallidus and can create a signal void in the pallidal portion of functional images.) This basal ganglia loop did not show increased activity for generation of pseudowords compared to rest, suggesting that recruitment of VA/VLa and the other components of this basal ganglia loop was due to the lexical demands of the word fluency tasks. It is worth noting that neither the Copland et al. (2000) nor the Crosson et al. (2003) study isolated lexical search procedure proposed in this paper; as a result, whether the proposed search procedure is responsible for their findings remains a question for future research.

Crosson et al. (2007) did describe a procedure by which the basal ganglia loop just described might influence word finding. This procedure invoked three sub-loops and was based on Nambu’s (2003) analysis of such sub-loops in motor function. We first discuss the functions of the direct and indirect subloops in word finding, each acting through the VA/VLa gateway to the cortex. The direct loop was thought to be responsible for enhancing the most probable word choice (e.g., the word most commonly associated with the semantic concept) while the indirect sub-loop suppressed competing alternatives (e.g., words less commonly associated with the semantic concept). This process should enhance the signal and decrease the noise in the word-finding process, thereby reducing the probability of errors in word selection. These concepts can be integrated with the concept of iterative searches of progressively smaller lexical-semantic neighborhoods. The cortico-cortical machinery described in the earlier section would work to convert a semantic concept, early on, to a lexical neighborhood of semantically related items and, with the help of the stable concept maintained by cortico-thalamo-cortical processing, confirm the neighborhood most commonly associated with the semantic concept. The job of the basal ganglia, then, is to enhance the activity of the most commonly associated lexical neighborhood while suppressing activity in the competing neighborhoods. This contribution of the basal ganglia increases the signal and reduces the noise in the lexical selection process, making it more efficient and less error prone. Once an appropriate lexical neighborhood has been selected, it is then probed for a smaller neighborhood of more highly semantically related items until the neighborhood consists of a few items from which the appropriate item is selected (see the German shepherd example in the previous section). At each iteration of the search, the basal ganglia’s contribution is to increase the signal-to-noise ratio, reducing the probability of errors. Also, as an extension of Crosson et al.’s (2007) analysis, the third (hyperdirect) basal ganglia subloop resets the process to begin a search of the recently selected lexical neighborhood, thereby moving the lexical selection procedure to its next phase. [The hyperdirect subloop bypasses the striatal and lateral pallidal components to directly access the subthalamic nucleus, which in turn, accesses the thalamus *via* the medial globus pallidus (Nambu, 2003; Crosson et al., 2007)].

Hence, we suggest that two mechanisms, one working through a basal ganglia loop and the VA/VLa thalamus the other acting through a cortico-thalamo-cortical mechanism and the pulvinar both play different roles in converting semantic concepts into words to represent them. One might ask how two such mechanisms, anatomically distinct at the level of the thalamus, can act in such finely tuned coordination to support word finding. The answer is through the mutual connectivity of these two mechanisms with anterior cortical language areas. In macaques, Goldman-Rakic and Porrino (1985) showed that the ventral anterior nucleus and the medial subnuclear sector of the pulvinar both are connected to nearly every prefrontal area. Since what we call Broca’s area in macaques is only nascent in macaques, we used tractography with diffusion weighted images to verify that both the dominant VA and the dominant pulvinar were connected to both the anterior (pars triangularis) and posterior (pars opercularis) portions of Broca’s area (Bohsali et al., 2015). Further, it has been shown that the anterior portion of Broca’s area is more involved in semantic than in non-semantic (phonological) processing of words while the posterior portion of Broca’s area is more involved in phonological than semantic processing of words (Devlin et al., 2003). Hence, the process of converting a semantic concept to a lexical item to represent it can be orchestrated by Broca’s area, assisted by VA/VLa and the pulvinar.

To summarize, in this subsection, we have considered the possibility that the act of word finding has a procedural as well as a declarative component. The procedural component involves how we search our lexicons for a word to precisely represent a concept that we have in mind. Like other forms of procedural memory it is employed without conscious deliberation to translate concepts into words. Further, this procedure relies on declarative knowledge about words and their meanings. In other words, the learned search procedures and the semantic-lexical (declarative) knowledge-base on which the search is conducted are so intimately intertwined that it is difficult to separate them. The role of the basal ganglia is to enhance the selection process at each stage of the iterative process by increasing the signal-to-noise ratio and to move the search process along from one stage to the next.

## Conclusion and Synthesis

In our final remarks, we will state four propositions based on the above analysis of thalamic anatomy, cognitive-linguistic sequelae of thalamic strokes (hemorrhagic and ischemic), and functional imaging studies. In the interest of guiding future research, some emphasis will be placed on the degree of certainty vs. the tentative nature of the conclusions. The fact that some conclusions are more tentative in nature indicates areas in which future research is necessary to validate and refine constituent concepts or perhaps to replace such concepts with conclusions better grounded in results from that future research. Given the somewhat infrequent nature of lesions primarily confined to the thalamus and the difficulties in designing functional imaging paradigms that can parse cortico-thalamo-cortical from cortico-cortical processing, it will be important to emphasize designs of future studies that can meet these challenges. Hence, one purpose of this section is to take a step toward such future research by clarifying what issues need to be resolved to understand the role of the thalamus in declarative vs. procedural linguistic processing. Our conclusions are as follows:

*Proposition 1: The pulvinar is an area of the thalamus involved in semantic processing which can be considered as declarative in nature.* Based on the nature of the evidence that we discussed, this conclusion can be considered to possess a relatively high degree of certainty. In particular, lesions of the posterior thalamus that encroach on the pulvinar, usually hemorrhagic, nearly always result in thalamic aphasia with the typical semantic paraphasias (semantically related word substitutions) that often deteriorate into jargon (Crosson, 1992). Recently, the author suggested that abundant semantic paraphasias in thalamic aphasias result from the loss of the thalamic ability to undergird the transformation from semantic concept to lexical item with a stable semantic representation which ensures that process of narrowing down lexical choices to more and more discrete neighborhoods of semantically-related lexical items stays on track (Crosson, 2021). This kind aphasia also can occur with ischemic lesions to thalamic regions anterior to the pulvinar, but as often as not, this kind of aphasia is not evident in these ischemic thalamic lesions (Crosson, 2021). As noted above, Bohsali et al. (2015) demonstrated that fibers from Broca’s area track through the thalamus in a position and orientation consistent with that of the internal medullary lamina, a white matter bundle traversing the thalamus from anterior to posterior (see Figure 1). These fibers can be disrupted by thalamic infarcts in both the polar and paramedian artery territories (e.g., Nadeau and Crosson, 1997). We concluded above that impingement of thalamic infarcts on the internal medullary may disconnect the pulvinar from anterior language cortices with a result similar to lesion of the pulvinar itself, i.e., prolific semantic paraphasias sometimes deteriorating into jargon. Further, it should be noted that fMRI studies of binding semantic features into object representations (Kraut et al., 2002a,b, 2003) are consistent with the idea that the pulvinar is involved in processing verbal and nonverbal semantic concepts, consistent with the interpretation of pulvinar function set forth in this article.

Some nuances of determining involvement of the internal medullary lamina during lesion mapping should be raised. Generally, mapping of thalamic lesions has occurred with standard clinical images and a camera lucida technique that can only estimate the exact location of thalamic nuclei and white matter based on subcortical atlases, such as that of Schaltenbrand and Bailey (1959) or more recently that of Morel (2007). But, techniques using inversion recovery MRI methods, such as the FGATIR sequence (Sudhyadhom et al., 2009) can image smaller subcortical white-matter bands, such as the internal medullary lamina, which would help determine if the latter has been damaged. Also, we have recently used the elastic thalamic atlas of Iglesias et al. (2018) to measure thalamic volume loss after nonthalamic stroke (Krishnamurthy et al., 2020) and believe that with additional work this atlas may be useful in more precise description of the topography of thalamic lesions.

*Proposition 2: The VA/VLa region is involved in a basal ganglia loop that supports word searches by enhancing activation for the chosen lexical items (or lexical neighborhoods) while suppressing activation of competing words (or lexical neighborhoods).* This hypothesis can be viewed as more tentative than the first proposition. The primary evidence for it comes from the Crosson et al. (2003) study cited above. In short subjects, showed activity in a dominant-hemisphere basal ganglia loop involving medial frontal cortex (pre-SMA/paracingulate cortex), the dorsal caudate nucleus, and the VA/VLa thalamic region when generating words from semantic categories or when generating words rhyming with a target word. They did not show such activity when generating syllables given beginning and ending consonant blends, suggesting that this basal ganglia loop is involved in the process of selecting words from a lexicon. This conclusion is in contrast to processes involving the dominant pulvinar, which appear to be more semantic in nature, as just noted. Indeed, after reviewing the literature on thalamic aphasia, Crosson (1992) reported that in some cases of thalamic infarction (anterior to the pulvinar) the prolific semantic paraphasias characteristic of dominant pulvinar involvement do not exist, consistent with a more general impairment of the word selection process. In other words, unlike the case of posterior thalamic lesion (or connections to the pulvinar), the problem is not with the inability to keep the necessary semantic concept active; rather, the problem is with selecting a lexical neighborhood or item at all.

More specifically, based on the work of Nambu (Nambu et al., 2000; Nambu, 2003) in the motor system, Crosson et al. (2007) suggested that direct portion of the basal ganglia loop from Crosson et al. (2003) was involved in enhancing activity of connections for representing the word selected for production and that the indirect loop (passing through the external globus pallidus and subthalamic nucleus in addition to the cortico-striato-pallido-thalamo-cortical connections of the direct loop) was involved in suppressing competing lexical alternatives. As applied to the iterative procedure for word finding above, we propose that this procedure can be applied to the early stages of word finding in which successively smaller and more relevant semantically-related lexical neighborhoods are selected during the search for the best word to represent a semantic concept. In other words, at each early stage of the search, a lexical neighborhood is selected for the next word-finding stage and competing neighborhoods are suppressed until a neighborhood is reached from which the best lexical item can be selected. Finally, a propos to the topic currently under discussion, is the question of whether this procedure represents a form of procedural memory. The author submits that this procedure represents a series of learned steps that are applied without conscious deliberation regarding the procedure. In that sense, the word-finding procedure can be compared to the procedure of selecting a syntactic frame or inflecting verbs, which Ullman (Ullman et al., 1997; Ullman, 2004) classified as a form of procedural memory. Whether the hypothesized procedure is or is not classified as a form of procedural memory, however, we must note that the evidentiary support for the proposition of involvement of VA/VLa is limited. Hence, further research is necessary to determine the validity of the hypothesis.

*Proposition 3: Through its connections with dominant frontal structures and the dominant basal ganglia, the dorsal medial thalamus may support syntactic and other grammatical operations.* The case for this proposition rests on a single case study, that of De Witte et al. (2006), which provided the most complete assessment of syntactic functions in a case of thalamic aphasia of which this author is aware. Problems were noted with both syntactic production and syntactic comprehension at 1month post onset. The authors attributed syntactic processing deficits to the facts that the dorsal medial nucleus is intimately connected to every prefrontal region (e.g., Rose and Woolsey, 1948; Leonard, 1969; Fuster, 1980) and that dorsal lateral prefrontal cortex plays a role in grammatical processing (e.g., Nadeau, 1988). Whether or not the reader accepts this syntactic processing as a form of procedural memory depends to some degree on whether s/he accepts the proposition that syntactic processing is a form of procedural memory as claimed by Ullman (Ullman et al., 1997; Ullman, 2004). It also should be noted that the dorsal medial nucleus is involved in basal ganglia loops that begin and end in the dorsal lateral prefrontal cortex (e.g., Alexander et al., 1986). Copland et al. (2021) suggested that through their relationships with the frontal lobe, the basal ganglia might play roles in domain general processes, such as working memory, that support syntactic and grammatical processing as well as domain specific processes that are syntactic or grammatical in nature. In this article, it was suggested that Giavazzi et al.’s (2018) concept of the basal ganglia being involved in selection of the best grammatical form would be consistent with previous analyses of both motor behavior (Nambu, 2003) and word finding (Crosson et al., 2007). Clearly, the evidentiary base regarding the hypothesis that the dorsal medial nucleus plays a role in syntactic and/or related grammatical processes needs to be expanded. To this end, investigators studying thalamic aphasias should add a variety of syntactic and grammatical tests to their assessment arsenals, and the De Witte (2006) offers a model of this type of assessment upon which future investigators can build. Carefully planned functional imaging studies may also be useful in determining if the dorsal medial nucleus plays a role in syntactical/grammatical processes, whether such a role is inclusive or independent of the basal ganglia, and what the nature of that role is, if indeed it exists.

*Proposition 4: The pattern of deficits after dominant thalamic infarcts will depend on which thalamic gray or white matter structure (or which combination of structures) is involved.* Inherent in our discussion to this point is the idea that there are brain systems involved in different linguistic processes. While these systems must communicate in every-day use of language and may even to a degree be overlapping (e.g., at the level of Broca’s area, as mentioned above), there also is a degree of topographic specificity to brain systems performing fundamentally different linguistic processes. Hence, we suggest that the cortical topography of processors necessary for a particular linguistic operation can be mapped onto the thalamus *via* thalamo-cortical and cortico-thalamic connections of these processors. This kind of systems approach is fundamentally different from the classic localizationist approach in that disruption of a linguistic function in a specific structure is not taken to indicate that the linguistic the function is located in that structure. Rather, it is an indication that the structure to which damage causes functional compromise plays a role in a system participating in the disrupted process. Functional imaging studies can help us to see the network of structures involved in specific linguistic processes. However, it takes thoughtful study design to unravel the nature of the contribution of different system components to a specific linguistic process in which they are involved.

In this article, we have proposed three kinds of processes in which thalamic nuclei may be involved. First, as noted above, we proposed that the pulvinar plays a role in semantic processing. That role is to stabilize activation of features constituting a semantic concept during an iterative word-finding process. This role involves connectivity to structures involved in both semantic and lexical processes, including the anterior and posterior components of Broca’s area, which are connected to the pulvinar *via* the internal medullary lamina of the thalamus (Bohsali et al., 2015). Hence, when the pulvinar or internal medullary lamina are damaged stabilization of the semantic concept is compromised, resulting in semantic paraphasias sometimes so prolific as to deteriorate into jargon. Second, the ventral anterior nucleus, *via* its position in a pre-SMA/pararcingluate cortico-caudate-pallidal-VA/VLa-pre-SMA/paracingulate loop, plays a role in an iterative search procedure in which increasingly specific semantic neighborhoods of lexical items are selected over competing neighborhoods until an appropriate lexical item to represent the intended semantic concept can be selected from a limited number of competitors [see Crosson et al. (2007) for a detailed description of the loop and procedure]. We have suggested that this iterative search process may represent a form of procedural memory acquired very early in life and that this procedure works very closely in concert with declarative memories to find the correct lexical item from a massive lexicon to represent the intended semantic concept. Such a close relationship between the declarative memory for what words represent and the search process that locates appropriate lexical items to represent semantic concepts would certainly be a challenge to dissociate in experimental paradigms. Severe disruption of this procedure by lesion would not result in semantic paraphasias but an inability to make any word selection at all. Third, based on a single case study (De Witte et al., 2006), we have suggested that the dorsal medial nucleus may be involved in syntactic and grammatical processes, either directly through the relationship between this thalamic nucleus and dorsolateral prefrontal cortex or indirectly *via* a basal ganglia loop with the same cortex. The latter two propositions have a more tenuous empirical base than the first and, therefore, require further empirical evidence.

Nonetheless, if one applies the above propositions and a knowledge the nuances of ischemic thalamic lesions, one can explain much of the variability in aphasia that they entail. To do so, one must understand that ischemic thalamic lesions are not topographically invariant. Although infarcts for a specific thalamic arterial territory are in the same general locale from one patient to the next, they do vary in size and shape. Further, thalamic nuclei are not homogenous in their cortical or subcortical relationships. Rather, there are subnuclear areas with specific projection patterns. Hence, important areas within a nucleus, particularly those toward the boundaries of an arterial territory, may show differences from one patient to the next in terms of whether they are included within the lesion boundaries of that artery. This kind of analysis of ischemic thalamic lesions is not new but has been around for at least three and a half decades. Specifically, von Cramon et al. (1985) showed that when infarcts of the dorsal medial nucleus subsumed the mammillothalamic tract, which courses through the nucleus, memory deficits were more profound than when this tract was spared. Bilateral infarcts subsuming the mammillothalamic tract caused more profound memory impairments than unilateral lesions. This pattern relating damage of the mammillothalamic tract in paramedian artery infarcts was confirmed by Graff-Radford et al. (1990).

We can apply this knowledge to dominant polar and paramedian artery infarcts to begin to understand the variability they cause in aphasia symptoms. For example, when dominant polar artery lesions damage the most posterior portion of VA/VLa, which participates in the basal ganglia loop described above, this damage will result in difficulties with selecting words, though not in prolific semantic paraphasias. However, given its location near the posterior boundary of the polar artery territory, it can be expected that in some patients this area will not be damaged. The internal medullary lamina, on the other hand, course near the medial border of polar artery lesions. If the more posterior portion of VA/VLa is not damaged but the internal medullary lamina is, then connections between Broca’s area and the pulvinar will be interrupted and the classical pattern of semantic paraphasias will be seen. If both the posterior portion of VA/VLa and the internal medullary lamina are damaged, word searches may not proceed far enough into the iterative search process to generate semantic paraphasias.

Similarly, Goldman-Rakic and Porrino (1985) showed that there is a relatively small and discrete region of the dorsal medial nucleus that projects to dorsal lateral prefrontal cortex in macaques. If this area in a human is damaged in a dominant paramedian artery infarct and the assumptions from De Witte et al. (2006) single case study are correct, then we might expect syntactic/grammatical deficits, but such impairments may be absent when dorsal medial thalamic communication with dorsal lateral prefrontal cortex is not affected. Further, when paramedian artery lesions involve the internal medullary lamina on the lateral periphery of its territory, communication between anterior language cortices and the pulvinar may be interrupted, again leading to semantic paraphasias. When communication between the dorsal medial nucleus and the dorsolateral prefrontal cortex and communication between the pulvinar and language cortices both are interrupted, we might expect both syntactic/grammatical deficits and prolific semantic paraphasias, as in the case of De Witte et al. (2006). However, since frequently neither of these deficits is reported in dominant paramedian artery lesions, it is possible that neither type of damage may be a common occurrence in dominant paramedian artery lesions.

Although the four propositions just considered relied heavily upon the literature on thalamic lesions (hemorrhagic and ischemic), there is a place for fMRI in studying involvement of thalamic subnuclear regions in various the linguistic networks. For example, the work of Kraut et al. (2002a,b, 2003) was cited above as contributing to our knowledge about the role of the pulvinar in semantic knowledge which was noted to be involved in linguistic declarative memory processes. The fMRI work of Crosson et al. (2003) also was cited as evidence of a role for the basal ganglia and the VA/VLa thalamus in word retrieval processes. Carefully designed neurolinguistic fMRI paradigms may be useful in unraveling the thalamic topography of proposed processes described above, especially if new techniques such as inversion recovery MRI (e.g., Sudhyadhom et al., 2009) and the elastic thalamic atlas of Iglesias et al. (2018) are applied to assist with identification of thalamic topography. However, the iterative nature of word retrieval processes as discussed above and by Crosson (2021) will require techniques with higher temporal resolution, such as magnetoencephalography, electrocortography, or stereotactic electroencephalography to confirm the rapid iterative processes during word searches.

In summary, we have discussed potential involvement of thalamic nuclei and their subnuclear regions in linguistic declarative and procedural memory processes. There is a great deal of empirical support that the pulvinar has a role in semantic-lexical processes that are declarative in nature. The nature of this role, as laid out by Crosson (2021), was summarized. We discussed two potential roles for subnuclear thalamic regions in linguistic procedural memory. The first involves the role for the subnuclear region of the dorsal medial nucleus projecting to dorsolateral prefrontal cortex in syntactic/grammatical procedures, identified as a form of linguistic procedural memory by Ullman (Ullman et al., 1997; Ullman, 2004). The direct empirical support for this proposition relies mainly on a single case study by De Witte et al. (2006), although a considerable amount of evidence suggesting involvement the basal ganglia in syntactic and related grammatical processes and/or the domain general functions supporting syntactic/grammatical processes (Copland et al., 2021) implicates thalamic mechanisms since VA/VLa and dorsal medial nucleus components act as gateways between the basal ganglia and cortical mechanisms. Finally, perhaps the most controversial suggestion was that the posterior portion of VA/VLa was involved in a word-finding procedure acquired early in life as one begins to build a vocabulary substantial enough to require a mechanism for identifying a correct lexical item to represent a concept. We proposed that this also was a form of procedural memory, working with declarative memory semantic and lexical representations to ensure accurate lexical representation of the intended concept. This description of a basal ganglia loop active during word searches by Crosson et al. (2003, 2007) was the impetus for this suggestion, and it is, to a degree, supported by the lesion literature as well. We discussed the need for future research into the propositions outlined in this paper. In particular, the latter two propositions regarding word search and syntactic/grammatical procedures will be fertile ground for future investigations.

## Author Contributions

The sole author is responsible for reviewing the literature, formulating hypotheses about the literature, writing the manuscript and obtaining permissions for reproduction of the figures.

## Funding

Work on this manuscript was supported by a Senior Research Career Scientist Award from the US Department of Veterans Affairs Rehabilitation Research & Development Service, grant # B9270L. The views presented in this work do not necessarily represent the views of the United States Government or the Department of Veterans Affairs.

## Conflict of Interest

The author declares that the research was conducted in the absence of any commercial or financial relationships that could be construed as a potential conflict of interest.

## Publisher’s Note

All claims expressed in this article are solely those of the authors and do not necessarily represent those of their affiliated organizations, or those of the publisher, the editors and the reviewers. Any product that may be evaluated in this article, or claim that may be made by its manufacturer, is not guaranteed or endorsed by the publisher.
